# A case of persistent hepatitis E virus infection in a young adult with no medical history

**DOI:** 10.1002/ccr3.7217

**Published:** 2023-04-16

**Authors:** Kento Shionoya, Makoto Kako

**Affiliations:** ^1^ Gastroenterology Medicine Center Shonan Kamakura General Hospital Kanagawa Japan

**Keywords:** acute hepatitis, chronic infection, HEV, IgA

## Abstract

Most patients with hepatitis E virus (HEV) infection are asymptomatic and improve naturally without any treatment, but even non‐immunocompromised individuals may develop persistent HEV infections and should be monitored regularly for the onset.

## Graphical abstract

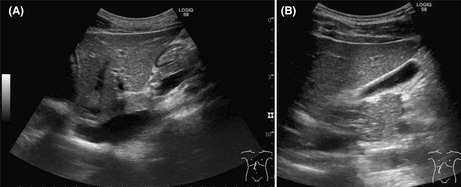

## 1 INTRODUCTION

Hepatitis E virus (HEV) is transmitted via the fecal‐oral route and can cause chronic infection in immunocompromised individuals. A 23‐year‐old man with no medical history visited our hospital after testing HEV positive during blood donation. Although he did not develop acute hepatitis, HEV IgA remained positive for over eight months.

Hepatitis E virus (HEV) is transmitted via the fecal–oral route and is particularly prevalent in young people in developing countries with poor sanitation, where it can occur sporadically and epidemically. Symptoms of acute HEV infection include general fatigue, slight fever, and nausea. Most patients with HEV infection are asymptomatic and improve naturally without any treatment. However, in immunocompromised individuals, HEV infection can become chronic. While there are many proven sources of infection, in some cases, the source of infection remains unknown. Recent studies have shown that HEV is a more common pathogen than previously thought, even in industrialized countries. In Japan, there have been few reports of chronic HEV infection in conventionally healthy people. We report a case of persistent HEV infection in an otherwise healthy young adult.

## 2 CASE REPORT

**FIGURE 1:**
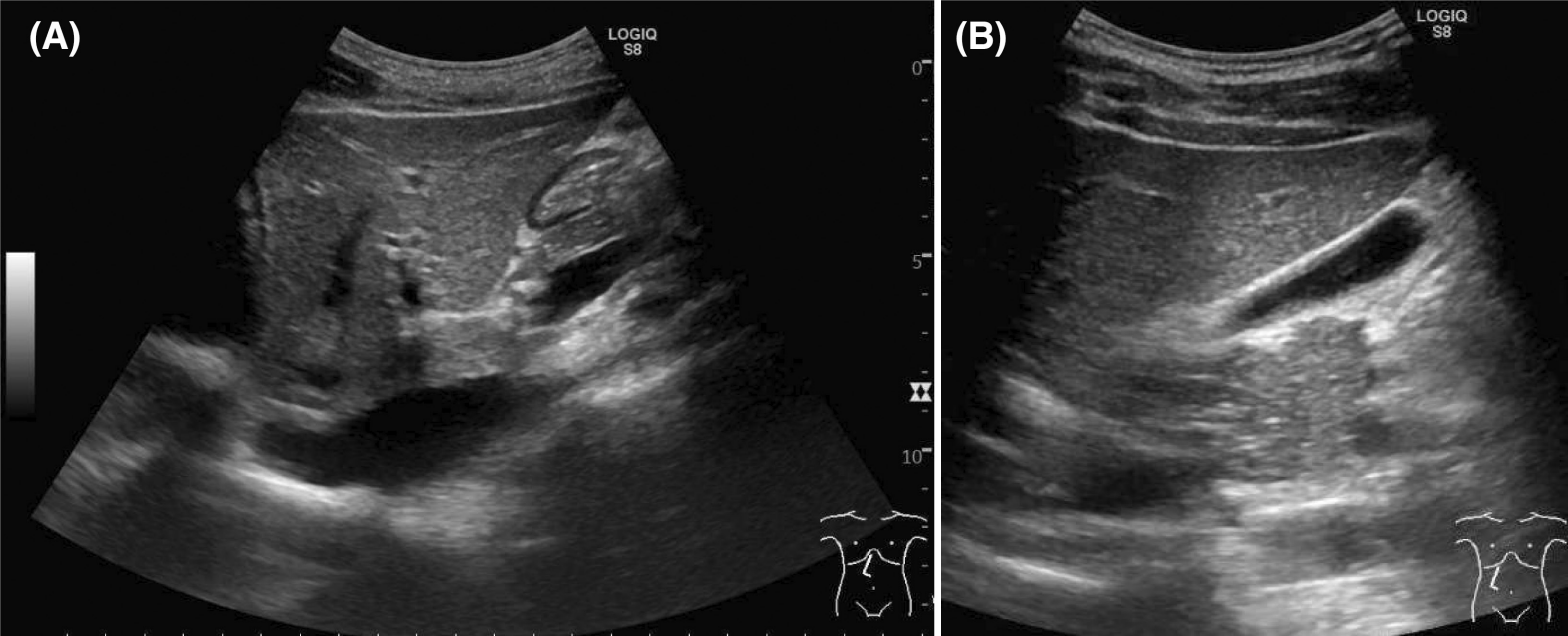
Abdominal ultrasonography. Abdominal ultrasonography showed no specific change.

A 23‐year‐old man with no medical history visited our hospital because his blood tested positive for HEV RNA at the time of blood donation. He had no history of smoking or drug use. His drinking was limited to occasional drinking. He had no symptoms, and laboratory tests did not show elevated hepatobiliary enzyme levels (aspartate aminotransferase, 18 IU/L; alanine aminotransferase, 8 IU/L; total bilirubin, 0.6 mg/dL); he had a normal white blood cell count of 6300/mm^3^ and a C‐reactive protein level of 0.017 mg/dL. He tested positive for HEV immunoglobulin (Ig) A. Tests for co‐infection with hepatitis B and hepatitis C were negative (Table 1). In addition, abdominal ultrasonography did not show any specific change (Figure 1). Neither the patient nor his family had a history of HEV infection. There was no obvious cause for the HEV infection, such as a history of international travel, surgery, or blood transfusion, and he had not consumed raw meat for 3 years. He showed no symptoms and was not diagnosed with acute HEV infection. The patient was followed up regularly in an outpatient clinic. There was no onset of clinical symptoms, and periodic outpatient blood tests showed no elevation of hepatobiliary enzymes, but HEV IgA remained positive for more than 8 months. The patient has been persistently infected with HEV and followed up on an outpatient basis, but he has not developed hepatitis.

**TABLE 1:** Laboratory data on the day of the first visit of the patient to our hospital.

Biochemistry			Blood cell count		
AST	18	IU/L	WBC	6300	/μL
ALT	8	IU/L	RBC	506	×10^4^/μL
LDH	180	IU/L	Hb	15.5	g/dL
γ‐GTP	12	IU/L	Plt	26	×10^4^/μL
ALP	64	IU/L	Coagulation system		
Cr	0.79	mg/dL	PT	101.8	%
BUN	14.6	mg/dL	PT‐INR	0.99	
T‐Bil	0.6	mg/dL	APTT	35.9	s
CK	227	IU/L	Virological examination		
Alb	4	mg/dL	HEV‐RNA	(+)	
CRP	0.017	mg/dL	IgA‐HEV	(+)	
Na	139	mmol/L	HBs Ag	(−)	
K	4.1	mmol/L	HBc Ab	(−)	
Cl	104	mmol/L	HCV Ab	(−)	

Abbreviations: γ‐GTP, γ‐glutamyl transferase; Alb, albumin; ALT, alanine aminotransferase; APTT, activated partial thromboplastin time; AST, aspartate aminotransferase; BUN, blood urea nitrogen; CK, creatine kinase; Cl, chloride; Cre, creatinine; CRP, C‐reactive protein; HBsAg, hepatitis B surface antigen; HBcAb, hepatitis B core antibody; HCV Ab, hepatitis C virus antibody; K, potassium; LDH, lactate dehydrogenase; Na, sodium; Plt, platelet count; PT, prothrombin time; PT%, percentage of prothrombin; PT‐INR, prothrombin time‐international normalized ratio; RBC, red blood cell count; T‐Bil, total bilirubin; WBC, white blood cell count.

## 3 DISCUSSION

HEV is known to cause acute hepatitis in developing countries, and regional outbreaks occur often.^1^, ^2^ HEV is an approximately 7.2‐kb single‐stranded RNA virus belonging to the genus *Orthohepevirus* of the family Hepeviridae. Genotypes 1–4 infect humans, with Types 3 and 4 reported in Japan, mainly in Hokkaido and eastern Japan.^3^ HEV causes zoonotic infections. Known sources of infection include venison, pork, boar meat, and contaminated water, but there are also reports that the consumption of wild plants such as blackberries and blueberries poses a risk for infection. Transfusion is another known risk factor for infection. However, in some cases, even with detailed interviews, the source of infection remains unknown.^4^ The incubation period is 2–10 weeks, but in most cases, the disease develops within 4–5 weeks after infection. Anti‐HEV IgM and IgG antibody levels rise during the incubation period. IgM is detected only for 3–12 months, whereas IgG can be detected for several years after infection.^5^ The symptoms of hepatitis include general fatigue, slight fever, nausea, and dark urine. HEV infection often resolves spontaneously, but caution should be exercised because it can become severe in pregnant women and immunocompromised patients and progresses to acute liver failure in rare cases.^4^, ^6^, ^7^, ^8^ Recent studies have shown that HEV is a more common pathogen than previously thought, even in industrialized countries. In the United States, the prevalence of anti‐HEV IgG antibodies among adults is 21.0%.^3^ In Japan, although there have been few reports of chronic HEV infection in conventionally healthy people, the proportions of subclinical infections and undiagnosed cases are thought to be high.^9^ Although anti‐HEV IgM is used as a reliable and sensitive marker of recent HEV infection, the specificity of the solid‐phase assay for anti‐HEV IgM has been questioned in some cases, particularly in patients with IgM rheumatoid factors in their serum that exert activity against the Fc portion of IgG directed to the HEV antigen and may elicit a false‐positive result.^9^, ^10^, ^11^, ^12^ Virus‐specific IgA has been detected during the acute stage of infection with hepatitis A or B virus.^13^, ^14^ HEV IgA has been detected in sera from patients with hepatitis^10^ and was included in the national health insurance scheme in 2011. Since then, the number of reports has been increasing.^9^

Chronic HEV infection has been reported in patients who have undergone organ transplantation. Lasting viremia in such cases has been mainly caused by immunosuppression due to the administration of immunosuppressive drugs or induced by specific HEV genotypes.^2^, ^15^, ^16^ Patient factors associated with higher disease severity and mortality include being male and having comorbidities, such as tumors, diabetes, liver dysfunction, respiratory disease, and renal dysfunction.^4^, ^6^ In the present case, the patient had no history of immunodeficiency, and there was no obvious cause of the HEV infection. RNA testing can reliably identify the virus, but it is not covered by health insurance in Japan; it is more practical to follow‐up with blood tests for liver dysfunction and HEV IgA to determine whether acute hepatitis or liver failure develops. A previous study showed that in some cases, negative results were obtained within a few dozen days of onset, whereas in others, there were positive results after 140 days; therefore, it is not clear how long a patient remains positive after onset.^9^ In the present case, the patient did not develop acute hepatitis; however, HEV IgA remained positive for more than 8 months. The clinical significance of long‐term persistently positive HEV IgA remains unclear, and more data are needed. The patient was young, had no comorbidities, and had a low risk of severe disease; however, careful follow‐up is necessary.

## 4 CONCLUSION

As the number of reported HEV cases has been increasing in developed countries, it is expected that more young patients with persistently positive HEV IgA will be reported in the future. The clinical significance of long‐term persistently positive HEV IgA remains unclear; however, careful follow‐up is necessary.

## AUTHOR CONTRIBUTIONS

**Kento Shionoya:** Data curation; investigation; visualization; writing – original draft; writing – review and editing. **Makoto Kako:** Conceptualization; supervision.

## FUNDING INFORMATION

The authors do not have funding to disclose.

## CONFLICT OF INTEREST STATEMENT

The authors declare no conflicts of interest.

## INFORMED CONSENT

Written consent for publication was obtained directly from the patient in accordance with the journal's patient consent policy.

## Data Availability

Dataset are available from the corresponding author upon request. No additional data are available.
